# Effects of aging on neural processing during an active listening task

**DOI:** 10.1371/journal.pone.0273304

**Published:** 2022-09-07

**Authors:** Abin Kuruvilla-Mathew, Peter R. Thorne, Suzanne C. Purdy

**Affiliations:** 1 Speech Science, School of Psychology, University of Auckland, Auckland, New Zealand; 2 Eisdell Moore Centre, University of Auckland, Auckland, New Zealand; 3 Faculty of Medical and Health Science, University of Auckland, Auckland, New Zealand; 4 Brain Research New Zealand, University of Auckland, Auckland, New Zealand; La Sapienza University of Rome, ITALY

## Abstract

Factors affecting successful listening in older adults and the corresponding electrophysiological signatures are not well understood. The present study investigated age-related differences in attention and temporal processing, as well as differences in the neural activity related to signal degradation during a number comparison task. Participants listened to digits presented in background babble and were tested at two levels of signal clarity, *clear* and *degraded*. Behavioral and electrophysiological measures were examined in 30 older and 20 younger neurologically-healthy adults. Relationships between performance on the number comparison task, behavioral measures, and neural activity were used to determine correlates of listening deficits associated with aging. While older participants showed poorer performance overall on all behavioral measures, their scores on the number comparison task were largely predicted (based on regression analyses) by their sensitivity to temporal fine structure cues. Compared to younger participants, older participants required higher signal-to-noise ratios (SNRs) to achieve equivalent performance on the number comparison task. With increasing listening demands, age-related changes were observed in neural processing represented by the early-N1 and later-P3 time windows. Source localization analyses revealed age differences in source activity for the degraded listening condition that was located in the left prefrontal cortex. In addition, this source activity negatively correlated with task performance in the older group. Together, these results suggest that older adults exhibit reallocation of processing resources to complete a demanding listening task. However, this effect was evident only for poorer performing older adults who showed greater posterior to anterior shift in P3 response amplitudes than older adults who were good performers and younger adults. These findings might reflect less efficient recruitment of neural resources that is associated with aging during effortful listening performance.

## Introduction

Active listening (e.g. in social settings) involves a complex interaction between *auditory* and *cognitive* processes that ultimately determines an individual’s success in recognizing speech [1]. At the auditory level, speech information passes through an array of cochlear and neural “filters” that decompose the signal into slow amplitude fluctuations, called the envelope, and rapidly fluctuating temporal information, called the temporal fine structure (TFS) [2]. TFS cues convey acoustic features of the target speaker based on the speech formant structure and hence, intact TFS encoding is important for detecting speech information in the presence of fluctuating background noise [2–5]. Envelope cues are also important as they contain information (e.g. prosody) that is important for speech recognition in noise [6, 7]. At the cognitive level, attentional abilities are important for focusing on the target speaker while simultaneously suppressing interfering acoustic information. Using fMRI, Wild et al. [8] showed that the auditory cortex and areas along the superior temporal sulcus are sensitive to stimulus-acoustic cues as well as the listener’s attentional state. Consistent with these findings, a recent study in young normal-hearing listeners showed that individual differences on a multi-talker listening task were better predicted by the participants’ selective attentional abilities and sensitivity to TFS cues than by other variables such as working memory span, age, and hearing thresholds [9].

Probing such interactions between auditory and cognitive processes has been crucial to understanding difficulties experienced by older people when listening to speech in adverse listening conditions [10, 11]. While listening in quiet conditions might be easy for many older adults with normal or near-normal hearing, listening in adverse conditions can be difficult due to limitations imposed by the incoming signal (interference by background noise and/or signal clarity) and/or by age-related changes in cognitive abilities [12]. In addition, perceptual learning, mental representations, memory, and attention functions become less efficient in older adults when speech is degraded [13, 14]. Thus, in older adults, successful identification of a target from a sound mixture will depend on the integrity of bottom-up sensory processing [15], without an over-reliance on top-down cognitive processes [16].

Reduced stimulus audibility due to elevated hearing thresholds is a major cause of listening difficulty with age (e.g. [17]). However, hearing thresholds alone do not explain the variance in auditory performance across older individuals [18–21]. Older adults with normal hearing thresholds can show deficits in measures of auditory processing, speech perception in noise, and selective attention [22, 23]. Hence, additional age-related changes, such as reduced sensitivity to TFS cues (e.g. [24]), reduced temporal precision in the midbrain [20], and the inability to control attentional processes, especially those involved in suppressing irrelevant information [25], are likely to contribute to the listening difficulties of older adults. It is difficult to tease apart such effects of aging on cognition and auditory processing from peripheral hearing loss and the resulting suprathreshold deficits, as the majority of older participants (>60 years) inevitably show some form of high frequency hearing loss [26]. In the current study we investigated the association between low-level encoding (TFS sensitivity) and high-level ability to use cognitive resources (selective attention) on auditory performance of older adults in noise, using strategies to minimize effects of peripheral hearing loss on stimulus audibility.

Drawing on a variety of electrophysiological techniques, a number of studies have probed the neural mechanisms associated with age-related declines in listening performance [27–31]. Evidence for enhanced cortical envelope encoding and its association with poor ability to segregate speech from background noise exists in older adults with (e.g. [30]) or without hearing loss (e.g. [32]). Also, early components of cortical event-related potentials (ERPs) are often larger in amplitudes and longer in latencies in older compared to younger adults [27–29]. Such exaggerated cortical responses are thought to be mediated by the combined effects of aging and peripheral hearing loss [27, 33–35] and have been attributed to imbalance in excitatory and inhibitory processes along the auditory pathway [36, 37]. As a result, older adults are less able to ignore distracting maskers while listening in adverse listening conditions.

As stated earlier TFS cues are very important to speech recognition in noise (i.e. supports unmasking) but relatively little is known about how TFS information is processed in the aging brain. Previous ERP studies have mostly explored aging-effects on speech in noise stimuli where acoustic cues (ENV & TFS) were preserved and unaltered (e.g. [38]). TFS cues are less accessible for older listeners and those with hearing loss, even when hearing thresholds at low frequencies are normal [39]. Only one ERP study, that we are aware of, has explored the effects of age as a function of degraded TFS cues [40]. They recorded ERPs (contingent negative variation, CNV) to TFS-degraded speech in young and older adults, during a selective listening task. The magnitude of CNV responses increased with decreasing acoustic information (less TFS cues) in both groups and this was attributed to increased listening effort from attention-allocation associated with task difficulty when TFS cues were reduced. Contrary to previous reports (e.g. [41, 42]), this study did not observe any age-related differences in CNV amplitudes.

Age-related difficulties in processing of acoustic cues (e.g. temporal speech cues) may be related to differences in attentional resource allocation associated with pre-/post- stimulus processing and response selection. While younger individuals show flexible reallocation of cognitive resources such as attention to repair degraded speech input, this capacity can be limited in older individuals [43, 44]. Reduced resource availability may lead to greater recruitment of additional brain areas (e.g. prefrontal cortex, PFC) to maintain adequate performance [45]. This has been explored using ERPs and active versus passive listening tasks. For example, Alain et al. [46] found that during passive listening (clearly detectable stimuli) and performance on a concomitant visual task, ERP amplitudes were significantly reduced in older compared to younger participants. In contrast, during active listening, ERP amplitudes were similar between groups, indicating that older participants reallocated cognitive resources to successfully perform the task during active listening.

There remains a limited understanding of the relative importance of behavioral predictors (e.g. [47]), encoding fidelity (e.g. [27]) and/or resource allocation (e.g. [48]) on speech processing abilities in older adults. The core of this present study is to link together three measures (TFS sensitivity, Attention Network Test, ERPs) to explain why older people have more difficulty than younger people in understanding meaningful auditory signals in the presence of background noise competition. The combination of tasks in a single study is novel and lends more explanatory power than earlier studies that have looked at these measures individually. We investigated whether a fixed [good] level of comprehension of spoken-digits in two-talker speech masker was linked to the ability to follow temporal fine structure of harmonic complexes, performance on a visual selective-attentional task, and to early- and late-latency ERP recordings (to the spoken-digits in two-talker masker). Cortical activity to intact and TFS-altered speech was examined by looking at the neural dynamics of the early and later latency time windows of auditory processing, during a selective listening task. The degradation was intended to induce performance variability among listeners and to reveal better and poorer performance groups. The combination of perceptual and physiological measures used here helped probe the role of TFS-degradation on cortical representation in younger and older adults and helped determine if this degradation resulted in neural compensation or processing inefficiencies (e.g. exaggerated amplitudes [29], frontalization of P3 responses [49] that differed between groups.

Listening to speech with reduced TFS cues may result in perceptual errors, following which listeners are forced to reallocate attentional resources to successfully comprehend auditory information. The timing precision of ERPs (milliseconds) allows one to examine the time course and mechanisms underlying such resource allocation and their relation to performance [50]. We used source estimation analyses to identify cortical activation patterns in younger and older adults, to investigate age effects on neural strategies for supporting speech recognition with increasing listening difficulty during an active task. Explorative analyses were performed to investigate whether cortical activation was related to listening performance. Additionally, stimulus-spectrum audibility was adjusted to account for the high frequency hearing loss in some of the older participants [51]. Finally, we utilized an unbiased data-driven approach where activity from all the sensors were considered in the time-point-by-time-point comparison between listening conditions [52, 53].

## Methods

All methods and procedures were approved by the University of Auckland Human Participants Ethics Committee. Participants were fully informed of all procedures and provided their informed consent prior to participation in the study. We first conducted a pilot study on eight adults (data not included in the main study) to determine the optimal ERP (EEG) recording and analysis parameters, total testing time and practice trials required for completing behavioral and EEG tasks. Guided by this pilot study, we decided to conduct behavioral and EEG recordings for the main study on different days.

### Participants

Thirty-two older adults (OAs) aged between 60 and 74 years (*M* 69.2 years, *SD* 3.4) were recruited from the local community (56% females). Of these participants, two (females) were excluded from the analyses because of incomplete test data and poor performance (> 3 SD from mean scores of YAs) on the behavioral task. All other participants reported a healthy lifestyle, and no history of neurological or psychiatric illness. Twenty-three healthy young adults aged between 21 and 27 years (*M* 23.1 years, *SD* 1.8) served as the control group (91% females). The data from three young adults (2 females, 1 male) were excluded from the analyses due to excessive EEG artifacts (≥ ±150 μV). The final sample comprised data of 30 older (*M* 69.1 years, 1 left-handed) and 20 younger (*M* 23.0 years, 2 left-handed) adults, respectively. All participants in the older (9 bilinguals) and younger (10 bilinguals) groups were native New Zealand English speakers. All participants had self-reported normal or corrected to normal vision with glasses or contact lenses. Participants in both groups had 12 or more years of education. None were receiving medication (anti-depressant/anxiety) deemed likely to affect EEG. All participants were compensated per hour for their participation in the study. Although none of the OAs were screened for mild cognitive impairment, it is important to note that all were living independently and none of the participants in the OA group performed poorer than three SD from the mean-scores of the younger participants on any of the behavioral measures described below.

### Audiometry

Pure-tone air conduction thresholds were obtained for all participants at octave frequencies from 250 to 8000 Hz (GSI-61, Headphones: TDH39) (see Fig 2). The mean pure-tone threshold (0.250 to 8 kHz) averaged between ears (L/R) for OAs was 20.3 dB HL (SD = 7.3, Range: 10.1–41.3 dB HL). Eighteen OA participants had thresholds ≥ 20 dB HL at or beyond 4 kHz indicating some (mild-severe) high frequency hearing loss [21]. Three participants in this group were frequent hearing aid users for age-related hearing loss (~ 2.5 years of consistent use), but did not wear their hearing aids during testing. These participants were also included as previous studies have shown that hearing aid experience did not alter the ERP components significantly [51, 54]. In addition, the EEG findings (topographical analyses) were replicated with the three participants excluded and hence we chose to include them here so as to not lose statistical power. Stimulus-spectrum audibility was adjusted for OAs with hearing loss (see *EEG*: *Signal audibility)*. YA listeners had thresholds (0.250 to 8 kHz) better than 15 dB (Mean 5.3, SD = 3.4, Range: 4.3–6.3 dB HL). In both groups, inter-aural hearing asymmetry was not greater than 15 dB at any of the frequencies. All participants had normal tympanograms (GSI TympStar), showing no evidence of middle ear abnormality.

### Temporal processing

Sensitivity to monaural TFS cues was assessed using the TFS1 test [55]. The test was installed on a laptop and stimuli were presented via Sennheiser HD 25 1-ii headphones in a double-walled sound-attenuating booth. This test has previously been shown to correlate with speech recognition thresholds in noise and with age [4, 21]. The test utilizes an adaptive two-interval forced-choice decision task, with trial-trial feedback. Participants discriminate a harmonic complex tone (A) from an inharmonic complex tone (B) in which the harmonics are shifted upwards by the same amount in Hz (ΔF). Both ‘A’ and ‘B’ tone complexes had an envelope repetition rate with equal fundamental frequency (F0), but differed in their TFS. The test F0 was 200 Hz and the corresponding center frequency was 2200 Hz. The lowest frequency in the passband was 1800 Hz. All other parameters were set to the test default values. This ensured that the harmonics in the passbands were mostly unresolved, consequently producing a weaker pitch percept which might be conveyed solely based on TFS information [56]. The ΔF was the manipulated variable, and it was initially set to 0.5*F*0. The ΔF varied from trial to trial according to a 2-down 1-up procedure, to estimate the value of ΔF producing 70.7% correct responses [57]. The overall level of the stimulus was set to 30 dB SL, based on absolute-threshold measurements for pure tones at the filter center frequencies. All participants completed a practice run to ensure they understood the task. Following the practice run, one run was completed for each participant in the better ear, which was determined based on the participant’s audiometric thresholds. Measurements were repeated if the software indicated high variability (*SD* 0.2) in performance. Test results have been shown to be stable for levels of 30 dB SL and higher [55] and comparisons between the left and right ears show no significant differences for adults with symmetrical hearing [21].

### Visual selective attention

Oberfeld and Kloeckner-Nowotny [9] showed evidence for both visual (flanker task) and auditory (backward-masked intensity discrimination) measures of selective attention to explain the same proportion of variance in speech-identification performance. Hence, we used a visual flanker task to measure selective attentional abilities that were independent of the individual’s auditory abilities. The short version of the visual attention network test (ANT; [58]) was administered using the Inquisit 4 software [59]. Distance between the computer screen and the participant was ~ 60 cm. The ANT is designed to measure efficiency of the orienting and executive control networks through the combination of cued (no cue, center cue, spatial cue) and flanker (congruent, incongruent) conditions. While orienting involves the ability to selectively attend and focus on the sensory information, executive control involves the ability to ignore distractors. The short form of the ANT was selected as assessment of the alerting network was not the focus of this study and this helped reduce participants’ cognitive fatigue. During the ANT task, participants are visually presented with a row of arrows and a fixation cross. Participants used a mouse button press to respond to the pointing-direction (i.e., left or right) of a central target arrow while ignoring the surrounding flanking arrows. Participants were instructed to respond as quickly and accurately as possible. The efficiency of the attention network was determined by calculating mean reaction times (RTs, msec) for correct trials as a function of cue and flanker conditions for each participant. Participants first completed a practice block (12 trials) with feedback and then one experimental block (120 trials) with no feedback. Participants were able to use glasses or contact lenses while performing this task.

### EEG: Data acquisition and stimulus presentation

The EEG recording was performed in an electrically shielded sound-attenuating booth. During the EEG recording session, participants were seated comfortably and instructed to fixate their eye-gaze on a stationary-cross displayed on a computer monitor. EEG data were acquired from 36 channels: Fz, FP1, FP2, F3, F4, F7, F8, FCz, FC1, FC2, FC5, FC6, FT7, FT8, Cz, C3, C4, T7, T8, CPz, CP1, CP2, CP5, CP6, TP7, TP8, Pz, P3, P4, P7, P8, Oz, O1, O2, M1 and M2, using a Compumedics Neuroscan Quick-Cap with sintered Ag/AgCl electrodes. Scalp electrode montage was based on the international 10/10 system. Vertical eye blinks were recorded with electrodes above and below the right eye and a ground electrode was placed anteriorly on the midline, between FPz and Fz electrodes. A midline electrode placed between the Cz and CPz electrodes served as the online reference channel. Impedances were kept below 5 kΩ using traditional skin aberration method. In a few instances (e.g. high impedances despite skin prep, or thick hair), we adopted the SurePrep method as an alternative to obtain low skin potential values [60]. Signals were amplified via a SynAmps RT system, filtered online with a 400 Hz low-pass, and digitized at a sampling rate of 1000 Hz. The configuration and number of EEG channels were based on previous studies that have decomposed spatio-temporal components associated with auditory target detection [61, 62]. This 36-channel array also allowed us to speed the electrode placement process, prevent bridging between channels, and record good quality data. As spatial resolution was based on a 36 -channel array with a 10–10 montage, localization inaccuracies are possible. A recent study, using a simulated EEG source, showed that localization error distance with 32-channel recording mostly varied between 1.45 and 3.38 mm [63]. While high-density recording channels are preferable, studies have equally suggested the importance of recording good quality data (low noise) and report that low-density EEG recordings (<32 channels) can also reveal valuable information of source activation especially in the presence of focal neural activity [64].

Source activation differences between conditions in the current study were estimated using robust statistical procedures, which included corrections for multiple comparison. In addition, significant activations were validated with additional (between-groups) comparisons, as well as with reference to corroborating findings within existing fMRI literature. Resting-state EEG data and frequency following responses were recorded in some participants but not considered for this analysis. These measurements were done after completion of the experimental procedures described here. Stimulus presentation was controlled in the Gentask module of the Compumedics Neuroscan Stim2 system. To bypass delays associated with the stimulus presentation computer and accurately measure trigger timing at the onset of the stimulus, a Cedrus StimTracker was used to generate trigger pulses at stimulus onset. All stimuli were presented binaurally via mu-metal shielded ER-3C insert earphones (Etymotic Research).

### EEG: Stimuli and task

Stimuli used in this study consisted of spoken digits presented in competing background noise. Each monosyllabic digit (“One”, “Two” & “Ten”) was uttered by a New Zealand female speaker (mean F0 = 210 Hz) and recorded using an AKG HC 577 L omnidirectional headset microphone with pre-amp and Adobe Audition (AA) sound editing software (sampling rate, 44.1 kHz). From the original recording, digits were shortened to match in duration (370 ms) and were ramped on and off using a cosine function (4%). Inclusion of monosyllabic digits ensured that we could record artifact-free ERP components with adequate number of trials, without increasing the length of the recording session. The background two-talker babble was created using recordings of two female talkers reading semantically anomalous sentences [65]. The twenty sentences recorded from both speakers were concatenated and mixed into a single file. Any loud and quiet sections in the audio file were edited using the AA compression function. Both speech and noise files were normalized to the same RMS level (-22 dB full scale). The two-talker babble was played continuously for the duration of each block/condition to avoid the speech babble being time-locked with the onset (evoking ERP activity) of the target digit presentation.

Procedures for acoustically degrading the digits and noise stimuli have been presented in detail previously (see [40, 66] for details). Briefly, both speech and noise stimuli were first decomposed into 16 channels using a gammatone filterbank, with center frequencies ranging from 0.08 to 10 kHz. Then the TFS information was altered by replacing it with an envelope-modulated sinusoidal tone above a cut-off value. In the current study, we used a cut-off value of 0.11 kHz, such that TFS information for all the channels below this value was preserved and at or above 0.11 kHz, it was degraded. The alteration of the natural cues in the stimuli made listening in the *degraded* condition less intelligible and cognitively demanding compared to the *clear* condition where the signals were unaltered from the original recordings.

For each block, participants performed a number comparison task (adapted from [40, 66]) while they listened to the stream of spoken-digits presented in a two-talker co-located speech masker (see Fig 2). The stimulus train consisted of three single-digit numbers (“One”, “Two” & “Ten”) presented in a pseudo-randomized sequence. Participants were required to indicate if the target number (“Two”) was smaller or bigger than the previous number (“One” or “Ten”) in the stimulus train (with a two-alternative, smaller/bigger keypress). Participants made their responses with a response pad (Cedrus) placed on their lap, using the left thumb for “smaller” and the right thumb for “bigger”. Participants were instructed to listen for the target number and respond (smaller/bigger) as quickly and accurately as possible. Cues or feedbacks were not provided during this task. The task was performed under two listening conditions: *clear*, where the digits were easily discriminable from the noise (~100% correct recognition) and *acoustically degraded*, where the digits were less discriminable from the background noise (71% correct recognition), respectively. Participants completed this task after a session of familiarization using oral instructions and practice trials with feedback.

The rationale for using this task in comparison to a simple detection task was so that it reflected processes (auditory and cognitive) involved when listening to speech in complex environments. Only three digits were included in this closed-set number comparison task so as to reduce the memory load and assess the ability to direct auditory selective attention. During piloting younger participants found the task very difficult with the addition of more numbers and required many more practice trials. The task was developed with the intention that it could be used in future studies of other populations such as children with listening difficulties and adults with mild cognitive impairment. The addition of varying background babble during the active listening task ensured that the recognition process was challenging, and also representative of a challenging everyday listening task, within the constraints of a controlled lab environment. The task difficulty is reflected in the relatively slow reaction times obtained while participants performed the task during EEG measurements (see Fig 4). Reaction times to oddball stimuli (e.g. [67]) and n-back tasks (e.g. [68]) are often reported to be faster (~500 msec).

**Fig 1:**
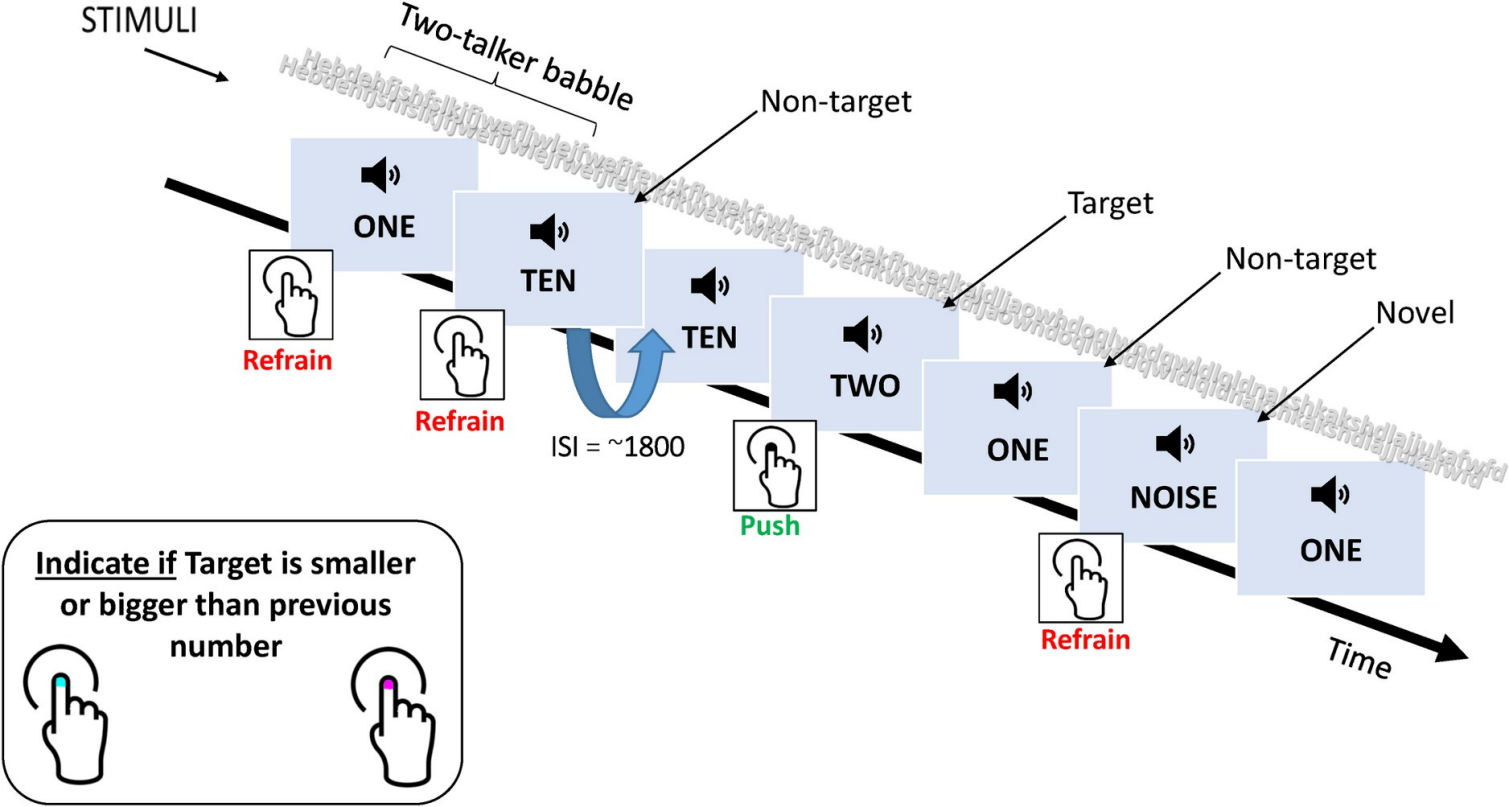
Example of task and sequence of events per trial, including time and auditory stimuli.

To record early and later stages of neural processing during the active listening task, the ERPs were elicited in the context of the three-stimulus paradigm [69]. Of the three digits, digits “one” and “ten” formed the non-targets, which occurred frequently (70% probability) and the digit “two” formed the target, which occurred infrequently (15% probability). An unexpected (novel), infrequent (15%) white noise stimulus (370 ms) was also presented, randomly interspersed among non-target stimuli (see Fig 1). The novel white noise stimulus was included to investigate neural indices (e.g. P3a ERP) of distraction/novelty processing and age-related decline of inhibitory processes during the selective listening task [70]. Therefore, this paradigm allowed us to study the effects of salient, but task-irrelevant distractors (involuntary attention) while participants selectively focused on the task-relevant information i.e. number comparison (voluntary attention). The data for the novel stimulus will be discussed separately (paper in prep.). Targets did not occur consecutively or at the start of a block. For each listening condition, two blocks of 250 stimuli (non-targets = 175, targets = 37, novel = 37) were presented. The clear and degraded listening conditions were counter-balanced across participants to reduce potential test order effects. To reduce temporal predictability, stimuli were presented in a random order with an inter-trial interval sampled at a uniform distribution (2203.39 ms ±100). Before the experimental EEG recording participants were presented with a brief practice trial of the number comparison task. Total recording time was ~34 minutes (excluding breaks).

### EEG: Signal audibility and adjusting for task performance level

Both procedures described below were completed on the first day of testing. These adjustments helped reducing the confounding effects of inter-individual differences in stimulus-spectrum audibility and recognition performance within each group. Controlling for stimulus-spectrum audibility is especially important as it accounts for some of the variances observed in the neural response of OAs with and without hearing loss [51].

Hearing loss in some participants would reduce stimulus-spectrum audibility and hence was compensated for using custom scripts (MATLAB) based on the NAL-R prescriptive formula for hearing aid amplification (National Acoustic Laboratories-Revised, [71]). Similar spectral adjustment using the NAL-R formula has been used previously in age-related research studies [47, 72]. Audibility/spectrum adjustments were performed for 18 of the 30 participants in the OA group who had some hearing levels of 20 dB HL or more. Signals (digits and babble) were amplified according to the participants’ audiometric thresholds (250–6 kHz). The digits were always presented at 75 dB SPL (for both groups), and this was calibrated (RMS level) using a 2cc coupler for insert earphones (G.R.A.S 90AB calibration system). All participants reported the digits (in quiet) to be clear and audible, without any perceived discomfort following this adjustment. We did not observe any significant correlations between behavioral task performance, ERP amplitude (TFS1, SNR_71%_, N1 amplitudes) and high frequency hearing thresholds in the OA group (*p >* 0.05). Validation of these efforts to reduce audibility effects was further provided by the behavioral performance in the *clear* condition where all participants (older & younger) performed the task with ~ 100 recognition.

**Fig 2:**
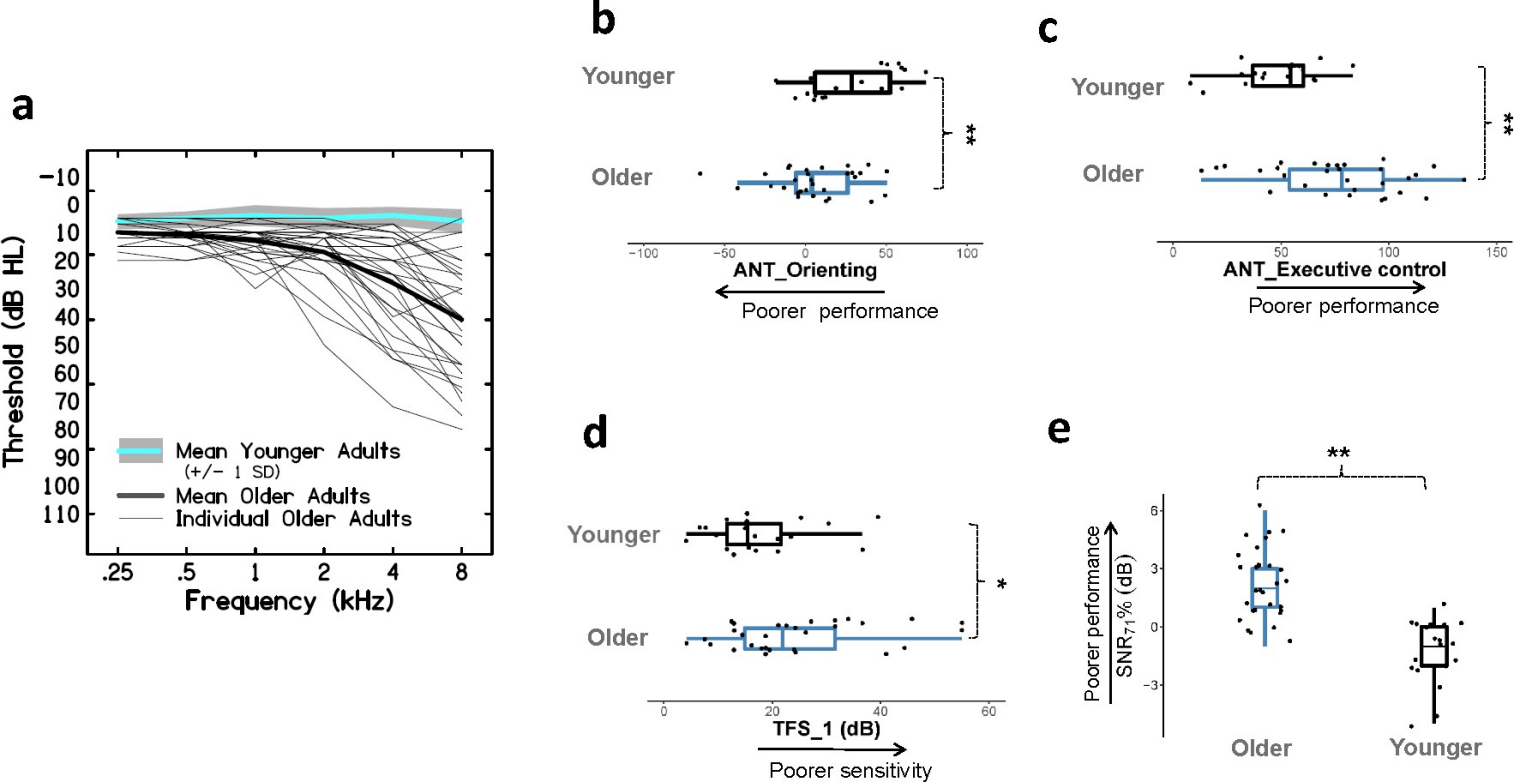
Behavioral measures. ***a*.** Pure-tone audiograms averaged between ears (L/R) for the Older and Younger participants. ***b*,*c*.** Performance on the attention network test (ANT): (c) Orienting network, (d) Executive control network. ***d*.** Sensitivity to TFS cues, using center frequency = 2.2 kHz in both groups. Poor performance on this task indicated poor temporal processing abilities. ***e*.** SNR_71%_ in the two groups measured in the digits-in-noise recognition task for the *degraded* listening condition. Poor performance on this task indicates poor visual sectional abilities. * *p < 0*.05 ** p < 0.*01*.

For each condition (*clear* & *degraded*), participants in the two groups were tested at the same performance level specific for that condition, ~100% recognition for *clear* and 71% for *degraded* speech. Performance scores were matched across all participants to ensure that results were not influenced by differences in task difficulty. This was achieved by first measuring the signal-to-noise ratio (SNR) that yielded a 71% correct score, i.e. SNR_71%_, in the *degraded* condition and then using this level to obtain ceiling-level performance (~100% correct) in the *clear* condition (SNR_*degraded*_ + 2 dB). Specifically, each trial started at an ‘easy’ 8–10 dB SNR (10 dB SNR for OA), and noise level was increased following two correct responses and decreased following each incorrect response (1 dB step size). Participants completed 30 trials in total. The arithmetic mean of the last six reversals was calculated to estimate the SNR yielding a 71% correct score [57]. Two threshold criteria, ~100% recognition for *clear* and 71% for *degraded* speech, were calculated for the participants in both groups. The SNR to achieve 71% in the *degraded* condition was also used as an individual outcome measure of speech understanding in adverse listening conditions (see Fig 2).

### EEG data pre- processing and ERP analysis

Offline processing of the EEG data was done using the CURRY 8 Neuroimaging Suite (Compumedics Neuroscan). Continuous EEG data files for each block were first concatenated into one file and then down-sampled to 250 Hz. Data were filtered (0.1–30 Hz) using a 2^nd^ order Butterworth IIR filter with zero-phase lag. Next, a threshold artefact rejection was applied (±150 μV) to remove any noisy segments. Independent component analysis (ICA), as implemented in EEGLAB (RUNICA, [73]), was used to remove ocular artifacts. The total number of removed artifactual ICA components per participant was on average 1.9 (Range 0–3) components for the EEG data. After the pre-processing, data were re-referenced to the average of all the channels (common average reference, CAR). Datasets were then segmented into 1100 ms epochs, starting 200 ms before each stimulus-onset. Data were baseline corrected relative to the 200 ms time window preceding stimulus-onset. Only epochs having the SNR thresholds (0.5–1.5 as implemented in CURRY) were included for averaging. In addition, only trials with correct responses to targets and without false positive responses to non-targets were included in the final averaging. Total trials included for averaging were similar across groups, listening conditions, and time-windows that were analyzed. For young and older adults, respectively, average numbers of accepted trials were: *Clear*: non-target trials: *M* 301.5 and *M* 227.2; target trials: *M* 55.8 and *M* 44.4; *Degraded*: non-target trials: *M* 292.5 and *M* 239.7; target trials: *M* 51.2 and *M* 38.2).

### ERP topographical analyses

Differences in topographies and underlying neural processes between the listening conditions were statistically tested using a permutation‐based nonparametric topographic analysis of variance (TANOVA, [74]). TANOVA identifies latencies of significantly different map topographies that are independent of the ERP magnitude. TANOVA is a global, unbiased statistical method that is not dependent on the choice of reference electrode or on predefined electrodes of interest or time-points to test differences in conditions. TANOVAs were performed using CURRY 8, which allows for within/across group comparisons to determine significant differences between conditions, latencies and, source analysis results. The analysis was performed on the non-target and target epochs separately with listening condition (*clear* & *degraded*) as a within-subject factor. This approach enabled us to investigate modulations of early and/or later stages of auditory processing for this acoustic manipulation of the stimuli. The multiple-comparison-corrected significance level was calculated based on the alpha level (0.05), the sampling rate of the data, and the low pass filter frequency [75]. This yielded a new alpha level value of 0.012 and randomization value of 4087, which further reduced false positive results. Only effects lasting for 20 msec or longer were considered significant. This approach has been shown to control for multiple comparisons and reduce false positives in mean global field power analyses [52, 76].

### Mean Global Field Power (MGFP)

The MGFP was calculated as the standard deviation of the amplitude data across all the EEG channels, reflecting synchronous activity at each time-point [77]. Similar to TANOVA, MGFP analysis helps summarize global brain activity and prevents the need to apply multiple testing correction on a subset of a priori electrode-sites that results in insufficient power. MGFP values were computed for each participant and each condition separately. Nonparametric randomization statistics for within-subject comparisons were performed using the Statistical Toolbox for Electrical Neuroimaging (STEN) developed by Jean-François Knebel (http://www.unil.ch/line/home/menuinst/about-the-line/software–analysis-tools.html). Statistical significance was obtained using 4000 permutations and an effect was considered reliable only for intervals where >20 ms contiguous significance was achieved (*p ≤ 0*.*01*).

### ERP source reconstruction

Information about the neural generators contributing to differences between conditions were estimated using the current density reconstruction (CDR) algorithm in CURRY 8 (Compumedics Neuroscan). First, an in-built head model based on the boundary element head method (BEM) was computed for each participant [78]. CDR was done using the standardized Low Resolution Brain Electromagnetic Tomography (sLORETA) method [79] to obtain current density images for the conditions compared. We only looked at current distribution that comprised the cortical grey matter and hippocampus in the MNI-referenced (Montreal Neurological Institute) brain. sLORETA estimates the underlying sources under the assumption that neighboring cortical regions should have synchronized and highly correlated neural activity. We used sLORETA because it has previously been shown to estimate (blurred) sources with minimal error when comparing it to fMRI-activations using an auditory oddball paradigm [80]. To test for significant differences in the source activation between conditions, CDR statistical nonparametric mapping (SnPM) was used [75]. This is a permutation or randomization test on the three-dimensional sLORETA images. As CDR SnPm extracts locations of significant source activation differences at every time-point, a correction for multiple testing was applied based on the spectral properties of the EEG data as described above. The resulting alpha level (0.012) and randomization value (4087) helped further reduce localization errors. Results of source localization analyses described in the next section include only that of the later time-window, as our initial analyses did not reveal any significant differences in source activation across conditions for either groups in the earlier time-windows of non-target stimulus processing.

## Results

### Effects of aging on behavioral measures

Fig 1 summarizes the results of all behavioral measures that were compared between the two groups. As the data were not normally distributed (Shapiro–Wilk Test, *p* < 0.05), non-parametric statistics (Mann–Whitney U-test) were applied for the analyses. The effect sizes were also computed using the following formula r = Z / √N [81]. For the TFS1 test, the OA group (Mean 24.6 Hz, SD 13.3) showed significantly poorer temporal processing abilities than YAs (Mean 17.8 Hz, SD 9.4), however, the effect sizes were small (*U* = 399.5, *p* = .049, *r* = 0.27). The OAs also exhibited significantly less efficient attention functions of orienting (*U* = 161.0, *p* = 0.006, *r* = 0.38) and executive control (*U* = 132.0, *p* = 0.001, *r* = 0.46) compared to the YAs, suggesting poorer selective attentional abilities (see Fig 2). The limitations imposed by poor temporal processing and selective attentional abilities in the OA group were also reflected in their performance on the digits-in-noise task. Noticeably, the OAs required higher SNRs (*M* 2.3 dB, *SD* 1.8) to achieve 71% correct performance compared to YAs (*M* -1.4 dB, *SD* 1.6). This difference was significant with a large effect size (*U* = 571.5, *p* < 0.001, *r* = 0.76). At the individual level, only five participants in the OA group had scores ≤ 0 dB SNR_71%._

**Fig 3:**
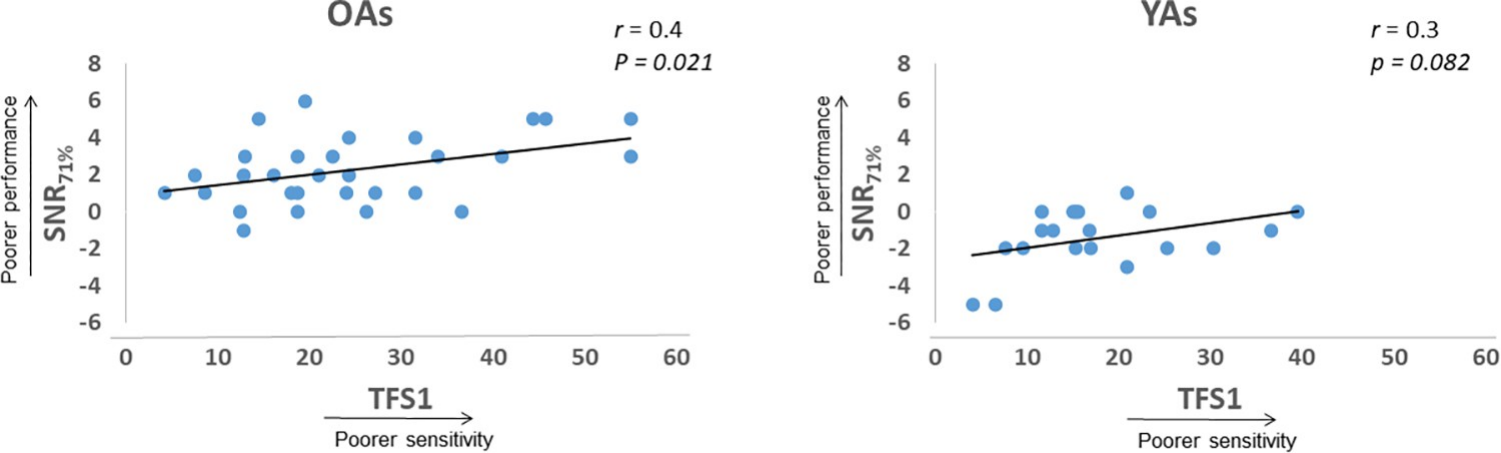
Scatter plots showing relationship between performance on the TFS1 task and the SNR_71%_ in older and younger participants.

To determine whether auditory (TFS1) and/or cognitive (ANT) abilities were predictive of their SNR_71%_ scores, a stepwise multilinear regression analysis was conducted separately for the older and younger adults [21]. To reduce the risks of overfitting and multiple testing errors associated with regression analyses, we performed a Bonferroni adjustment for the analyses (α = 0.025). Since the ANT scores for the orienting and executive control functions, measured on the same scale (msec), were correlated (*r* = -0.384, *p* = 0.006), the regression analysis was performed using the average of these two independent variables. For the regression analyses, the composite ANT and TFS1 test scores served as the predictors and the SNR_71%_ scores served as the dependent variable. For the OA group, the analysis indicated that SNR_71%_ scores were best predicted by a model only with the TFS1 scores (*R*^2^ = 0.17, *F*_(1, 28)_ = 6.02, *p*_*(corrected)*_ = 0.021). A scatter plot showing this association is shown in Fig 3. For the YA group, however, none of the variables significantly predicted the speech recognition scores (*p* = 0.048). Full model results for each group are shown in Tables 1 and 2 included in the S1 File. It is important to note that 18 participants in the OA group had age-related hearing loss and stimulus-audibility in these participants was individually compensated for using the NAL-R threshold correction. However, to ensure that addition of OAs with hearing loss did not affect the results of this analysis, we conducted a hierarchical regression analysis to investigate how much of the variance in the SNR_71%_ scores was accounted for by the hearing thresholds (250–8 kHz, averaged between ears) in OAs (see Table 3 in S1 File). The analysis revealed that, after removing the hearing thresholds, predictors (TFS1, ANT) in Model 2 accounted for significant variation (*R*^2^ = 0.19, *F*_(2, 27)_ = 3.35, *p* = 0.05). Hearing thresholds accounted for just 0.6% of variance in the SNR_71%_ scores. The most important predictor of SNR_71%_ scores was TFS1 scores, which explained 18% of the variance. This indicates that hearing loss may not have contributed to the entirety of the variation in the SNR_71%_ scores observed here.

### Early stages of auditory processing (non-target stimulus processing)

**Fig 4:**
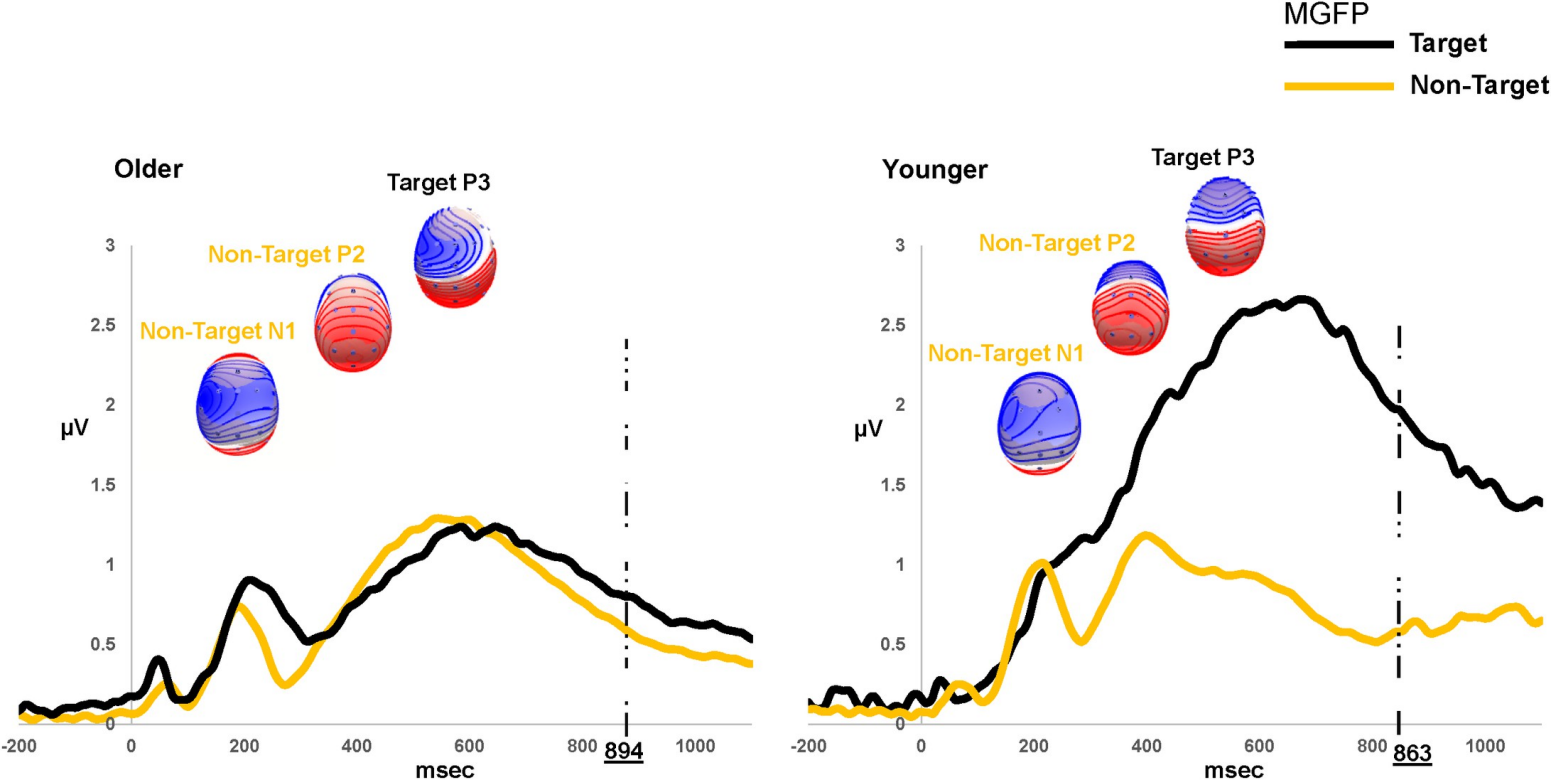
Grand-averaged mean global field power (MGFP) waveforms for the target and non-target stimulus processing for the older and younger participants. The corresponding scalp maps for the major ERP components; N1, P2 (Non-target) & P3 (Target), are displayed with positive values in red and negative values in blue. The dashed line shows the mean reaction time (averaged across conditions) for each group.

Grand average ERP waveforms and corresponding scalp topographies (averaged across listening conditions) for the older and younger adults are shown in Fig 4. Overall, non-target stimulus processing elicited a conventional P1-N1-P2 complex with the P1 component occurring at ~ 65 msec (Range 53–90 msec), N1 at ~202 msec (Range 165–296 msec), and P2 component at ~ 490 msec (Range 375–540 msec) (based on visual inspection of grand averaged waveforms). These latency intervals for the P1-N1 components are consistent with previous reports on ERPs recorded to speech stimuli in noise (e.g. [82]). The P2 component, however, appears later and temporally overlaps with the P3 component. This can be attributed to the paradigm/task used here, because participants had to make an internal decision (smaller/bigger) about the correct response, each time they heard the non-target number (“1” or “10”). Each response was thus prepared and withheld until the onset of the target number (“2”) and hence it is plausible that P3 overlaps with the P2 component during non-target stimulus processing. Time windows for TANOVA and MGFP analyses encompassed a broad latency range between 0 and 600 msec, which included the latencies of the early P1-N1-P2 complex based on the grand averaged waveform (see Fig 4).

#### Topographical analyses

**Fig 5:**
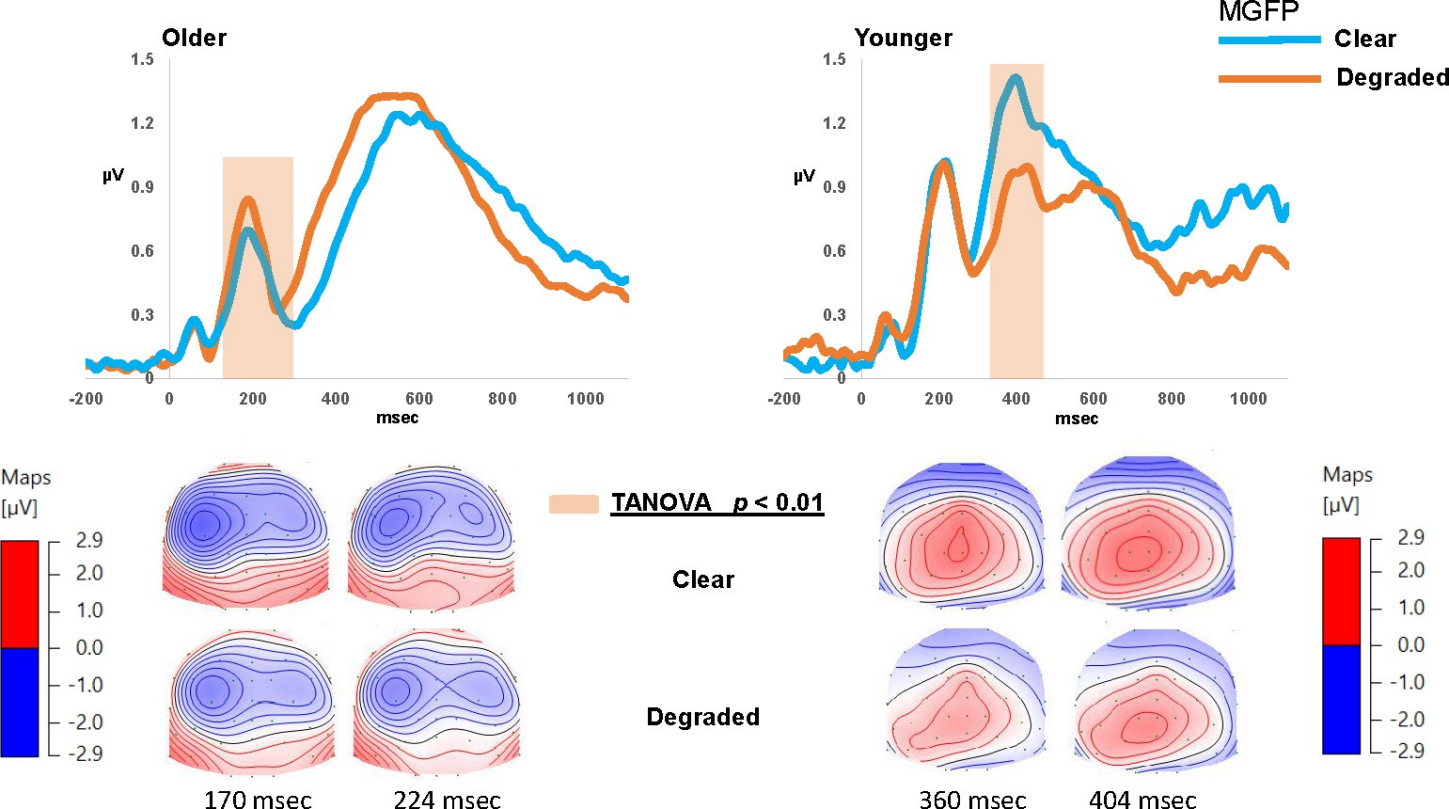
Topographical comparison of the non-target stimulus processing (epoch = 0–600 msec) for the older and younger participants: The top panel shows the grand-averaged mean global field power (MGFP) waveforms for the *clear* and *degraded* listening conditions in each group. Timeframes in the shaded region indicates periods of statistically significant differences (*p* < 0.01) between listening conditions. The bottom panel shows the corresponding topographical maps that are linked to the shaded timeframes of the MGFP for each listening condition.

TANOVA analysis comparing listening conditions was applied to the ERPs time-locked to the onset of the non-target stimulus from 0 to 600 msec. In the OA group, TANOVA revealed significant topographical dissimilarities in the latency ranges of 140.0 to 176.0 msec, 220.0 to 292.0 msec, and 316.0 to 368.0 msec, between the *clear* and *degraded* conditions (*p < 0*.*001*). From Fig 5 it is evident that differences in the listening conditions were most evident in the earlier, N1 latency range for the OAs. N1 (interval) topography showed largest negativity in the fronto-central electrodes with maximum activity over the left hemisphere. The topographies were stronger for the *clear* compared to the *degraded* listening condition. TANOVA in the YA group however, revealed significant differences in the topographies in the later intervals following signal onset between 312.0 to 324.0 msec, 332.0 to 384.0 msec, and 468.0 to 484.0 msec, (*p* < 0.001). Differences in the topographies between the listening conditions fell within the P2 range for the YAs. The P2 (interval) topography showed largest positivity in the centro-parietal electrodes with activity localized over the left hemisphere. The topographies were stronger for the *clear* compared to the *degraded* listening condition.

#### Mean GFP analyses

Mean GFP waveforms are displayed in Fig 5 in response to each listening condition and for each group separately. Between group amplitude differences (averaged between conditions) were statistically tested by extracting the mean amplitude of a 40 msec time window centered on each individual’s N1 or P2 peak latency (visually inspected from the grand averaged waveform). N1 amplitude was significantly smaller for the OAs (mean 0.94 μV SE 0.08) than the YAs (*M* 1.20 μV SE 0.09) (t _(unpaired)_ (48) = 2.39, *p* = 0.02, Cohen’s d = 0.6) but P2 amplitude was essentially identical between the groups (OAs 1.32 μV vs. YAs 1.13 μV) (*p* > 0.05). However, a time-point-by-time-point nonparametric test showed no MGFP difference in the post-stimulus intervals (0–600 msec) between *clear* and *degraded* conditions, for either groups (*p* > 0.05).

### Later stages of auditory processing (target stimulus processing)

**Fig 6:**
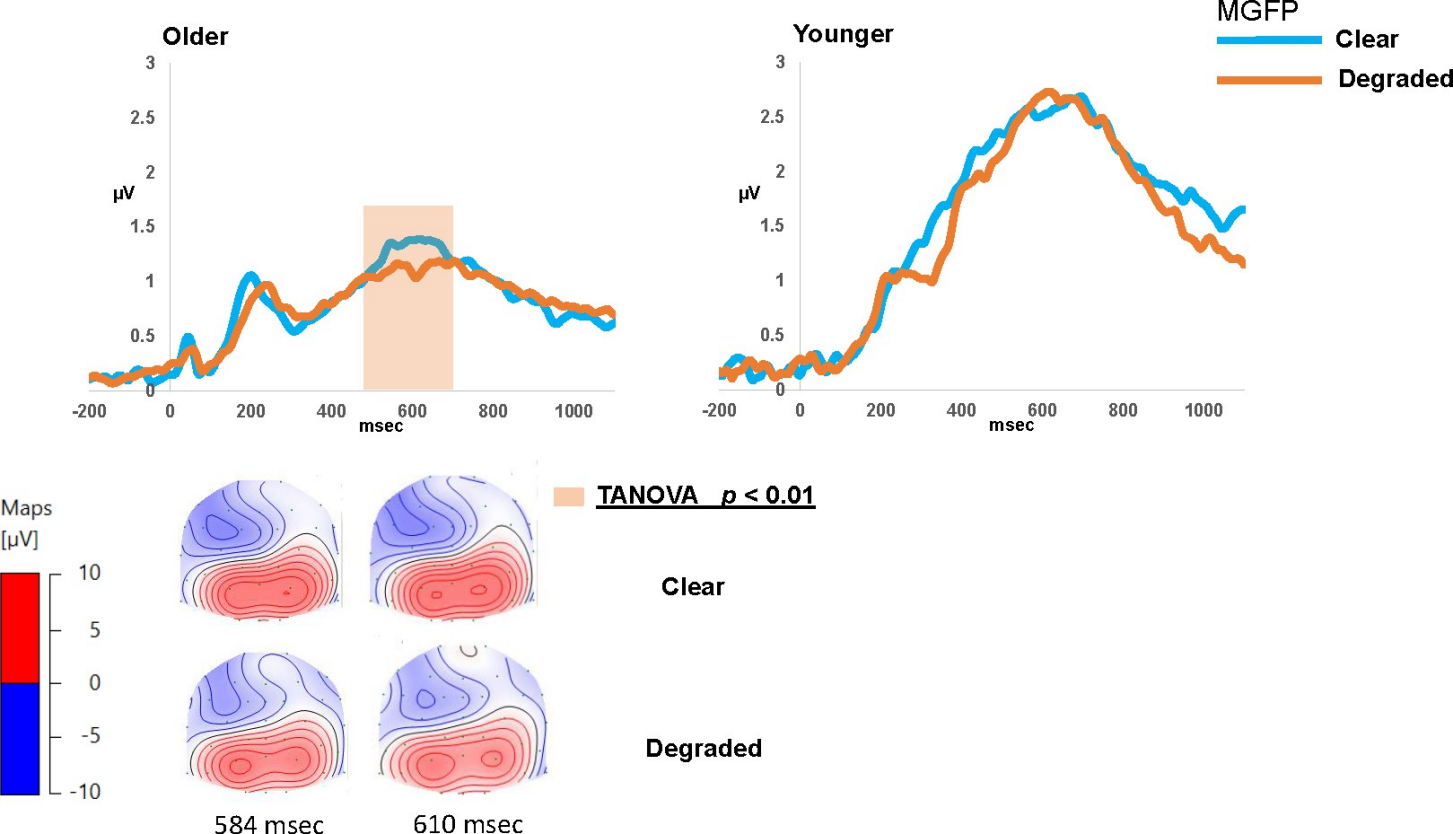
Topographical comparison of the target stimulus processing (epoch = 100–800 msec) for the older and younger participants: Top panel shows the grand-averaged mean global field power (MGFP) waveforms for the *clear* and *degraded* listening conditions in each group. Timeframes in the shaded region indicates periods of statistically significant differences (*p* < 0.01) between listening conditions. Bottom panel shows the corresponding topographical maps that are linked to the shaded timeframes of the MGFP for each listening condition.

Time windows for TANOVA and MGFP analyses encompassed a broad range between 100 and 800 msec, including the late P3 component based on the grand averaged waveform (see Fig 6).

#### Topographical analyses

TANOVA analysis comparing listening conditions was applied to the target-stimulus-locked ERPs from 100 to 800 msec (see Fig 6). For the OA group, the analysis revealed significant differences (*p* < 0.001) in the scalp distribution between the *clear* and *degraded* conditions in the latency ranges of 165.0 to 204.0 msec, 336.0 to 376.0 msec, 584.0 to 652.0 msec, and 680.0 to 764.0 msec. The P3 (interval) topography showed largest positivity in the parietal electrodes with maximum activity localized over the left hemispheres for the *degraded* listening condition and distributed over both hemispheres for the *clear* condition. The topographies were stronger for the *clear* compared to the degraded listening condition. However, for the YA group, no significant time window (≥20 msec) emerged in the *clear* versus *degraded* comparison (*p* > 0.01).

#### Mean GFP analyses

Mean GFP waveforms are displayed in Fig 6 in response to each listening condition and for each group separately. Visual inspection of the ERP grand averaged waveform shows that the P3 peak reached maximum values (μV) at ~ 610 msec after target-stimulus onset for both groups. Between group amplitude differences were statistically tested by extracting the mean amplitude of an 80 msec time window centered on each individual’s P3 peak latency (visually inspected from the grand averaged waveform). Mean GFP values (averaged across conditions) were larger for the YAs (mean 3.11 μV SE 0.32) compared to the OA group (*M* 2.34 μV SE 0.17) (t _(unpaired)_ (48) = -2.46, *p* = 0.01, Cohen’s d = 0.6). Within-subject comparisons of MGFP waveforms showed no significant differences between listening conditions for either group (*p* > 0.01).

#### Source localization analyses

**Fig 7:**
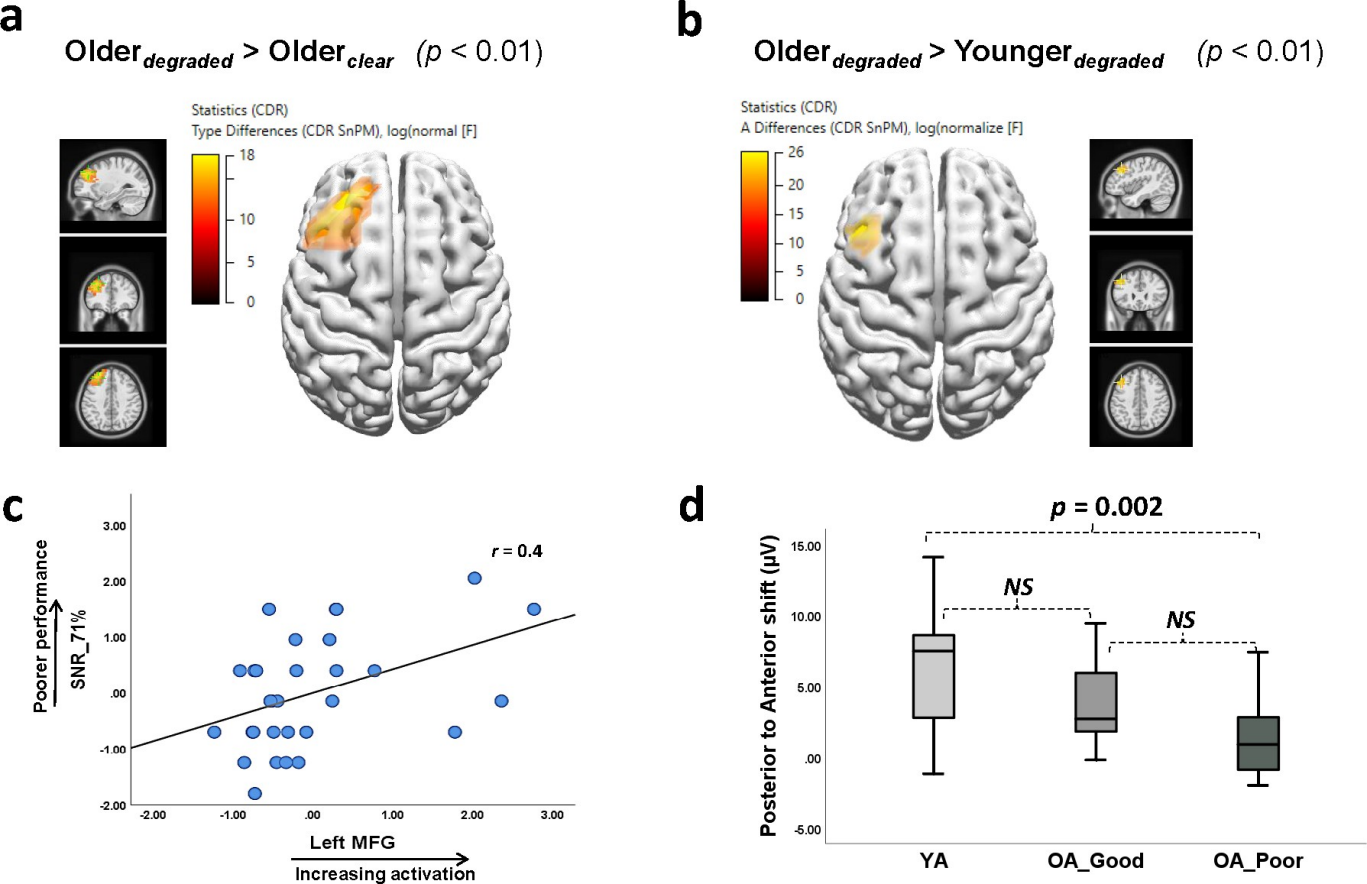
Top panel: ***a*,*b***. Displays results of the current density reconstruction statistical nonparametric mapping (CDR SnPM) testing. The input data for the CDR SnPM were the standardized Low Resolution Brain Electromagnetic Tomography (sLORETA) source images for the conditions compared. Source analyses revealed that Older participants recruited the left prefrontal cortex during degraded listening. Bottom panel: ***c*.** Scatter plot describing a significant positive correlation between scores on the digits-in-noise recognition task (SNR_71%_) and the left middle-frontal-gyrus activation in the Older participants (*r* = 0.42, *p* = 0.01). ***d*.** Displays differences in P3 amplitudes (Pz minus Fz electrodes) across younger (*n* = 20), good (*n* = 15) and poor (*n* = 15) older participants.

In general, source activation averaged across both listening conditions showed distributed activation patterns in the frontal, temporal, parietal, and limbic networks for both groups (> baseline [-200–0 msec], *p* < 0.01). Differences in brain activation were only computed for the time interval corresponding to significant effects of listening condition in the TANOVA analyses (distribution of scalp activity). Hence, statistical analyses of source estimations were computed only for the OA group (*clear* vs. *degraded*) in the time intervals between 350.0 and 764.0 msec. This interval showed significant differences in scalp distribution and comprised maximum P3 activity (see Fig 6). Results indicated significant differences in the ERP source activity in the frontal cortex (*p* < 0.01) of the left hemisphere with peak activity (degraded > clear) in the regions of middle frontal gyrus (MFG; Brodmann area (BA) 8, 9) followed by precentral gyrus (BA 6) (see Fig 7). To validate this source difference between conditions, additional sLORETA analysis for the P3 interval was computed between the two groups (YA vs. OA) for the *degraded* condition. The test for statistical significance (CDR SnPm) and multiple testing adjustments followed the same procedure as describer earlier. As expected, result of this analysis also showed an activation pattern of the prefrontal cortex (left MFG; BA 8, 9) during *degraded* listening in the OA group (*p* < 0.01, see Fig 7).

#### Brain-behavior relationship analyses

Exploratory analyses were conducted to evaluate whether this left MFG activation during *degraded* listening, in the OA group, was associated with their performance on the behavioral outcome measure. Local source activity in the left MFG was estimated using the scout function in Brainstorm toolbox [83]. Scouts were selected based on the Desikan-Killiany atlas and activity corresponding to the left rostral-MFG was used for correlational analyses. This resulted in one value (averaged over time) per scout and participant. Participants’ RTs and SNR_71%_ values, during the EEG task and scout values, respectively, were included in the analyses. Reaction times were of interest because meta-analyses of studies have shown improved RTs during working memory tasks following excitatory transcranial-direct-current-stimulation over the left dorsolateral prefrontal cortex [84]. Mean RTs in the OA group were significantly prolonged for the *degraded* condition (mean 938.6 msec) compared to the *clear* condition (*M* 850.1 msec) (t_(paired)_(29) = 5.81, *p* < 0.001) and compared to the YAs_(degraded)_ (*M* 862.3 msec) (t _(unpaired)_ (48) = 2.17, *p* = 0.03). We therefore investigated whether activation of the left rostral-MFG was associated with the speed of responding and/or accuracy in recognition. For statistical testing, raw values for each variable were transformed to z-scores before computing Pearson’s correlations.

Results indicated a significant positive correlation only between the left MFG activation and SNR_71%_, values (*r* = 0.42, *p* = 0.01). This finding was further supported by an increased (μV) posterior to anterior shift (Pz to Fz electrodes) in the ERP waveforms of the OAs compared to the younger participants [49, 85]. Specifically, the difference in P3 amplitudes (amplitudes averaged over ± 40 msec of peak, *degraded* condition) between Pz and Fz electrodes (representing the posterior to anterior shift) were compared between the YAs (*n* = 20) and two subgroups of OAs with good (*n* = 15) and poorer (*n* = 15) SNR_71%_ scores based on a median split. According to the median split, OAs with scores greater than 2 corresponded to poor performers, and those with scores less than 2 were good performers. Older participants with a score of exactly 2 (*n = 5*) were assigned to either group relative to their performance on the TFS1 test. SNR_71%_ scores between the independent groups (good vs. poorer) were not significantly correlated (*p > 0*.*05*), which reduces chances for Type 1 errors during median split analysis [86]. A one-way ANOVA with Tukey’s post hoc test revealed a significant difference only between the YAs and OAs with poor SNR_71%_ scores (*p*_*(Holm-Bonferroni corrected)*_ = 0.002). There was no significant difference between the OAs with good and poor SNR_71%_ scores (*p* > 0.05). Fig 7(d) shows an increased posterior to anterior shift with reduced amplitudes posteriorly (Pz minus Fz electrodes) in older poor-performers compared to good OA performers and younger participants (Poor<Good<YAs). Thus, in general, poorer performance was associated with a frontal shift in source activation during the degraded speech condition.

## Discussion

Here, we present a set of behavioral and physiological measures that helped identify potential predictors of speech recognition in noise and understand patterns of acoustic encoding and resource allocation in OAs vs. YAs, during a selective listening task. Behavioral measures of senility to TFS cues explained a significant proportion of the variance in the speech-in-noise recognition task (number comparison). Physiological measures revealed differential effects of aging on the dynamics of two latency time-windows during auditory-neural-processing. In addition, older participants showed source activation in the region of the left MFG for the challenging (*degraded*) listening condition. This source-estimated brain activity correlated with poor performance on the behavioral listening task. Poorer performing older adults showed greater posterior to anterior shift in P3 evoked response amplitudes than older adults who were good performers and younger adults.

Behaviorally, OAs showed limitations in temporal processing abilities with small effect sizes and significantly poorer (visual) selective attentional abilities (see Fig 2). Füllgrabe et al. [21] similarly showed reduced TFS sensitivity (TFS1 test), selective attentional abilities (Test of Everyday Attention), and working memory capacity in OAs compared to YAs (matched in mean pure-tone thresholds), using a smaller sample size. The smaller effect sizes observed in our study might be attributed to the center frequency (2.2 kHz) that was chosen to perform the TFS1 task. Performance on this task has been shown to improve with increasing filter center frequencies [55, 39]. Another possible reason may be our inclusion of an older adult group with hearing loss, whereas Moore and colleagues included a younger sample group (21–61 years) with normal hearing thresholds. Although, hearing loss can have an effect on task performance, only one out of the three participants in the older group with hearing threshold > 25 dB HL at 2 kHz performed the TFS1 poorly (44.4 Hz). In contrast, Schoof and Rosen [47] found reduced cognitive abilities (working memory, processing speed) in OAs with no differences in temporal processing abilities between groups (YA vs. OA). The opposing results between studies presumably reflect difference in task frequency shift detection (monaural TFS) vs. frequency modulation detection (binaural TFS) used to test temporal processing. Schoof and Rosen [47] also showed that performance on a speech-in-babble task was best predicted by inter-individual differences in TFS processing, rather than individual cognitive abilities. Interestingly, our regression analyses also showed TFS sensitivity to be predictive of performance in the *degraded* listening condition, only for older participants. Together, these findings suggest the importance of having intact sensory (auditory) processing, especially for OAs, while performing a cognitively challenging speech-in-noise task. Although the behavioral tests used in the current study do not probe temporal and/or attentional abilities extensively, they are commonly used laboratory measures and the results do support earlier findings that TFS sensitivity is important for understanding degraded information [47], and these abilities are less efficient in OAs [39].

### Electrophysiological differences at earlier latencies

Despite adjusting for frequency-specific stimulus audibility, OAs showed differences in early sensory processing corresponding to the time-window of the N1 potential. This may be indicative of deficits in supra-threshold auditory processing rather than hearing acuity that is commonly associated with aging [87]. Aging effects observed in the current study for early (~140 ms) stages of neural processing are consistent with reports of age-related neural degeneration beginning at the level of the auditory nerve fibers (in animal studies, [88]) and reduced neural precision at the midbrain level [20, 29]. Primary afferent deficits observed in older experimental animal studies appear to involve high-threshold neuronal fibers, which determine performance on supra-threshold auditory tasks such as detection of signals in noise and speech coding, rather than sound detection. Early neural processing deficits such as this may account for some of the age-related effects on cortical ERPs and behavioral performance observed in the OAs of the current study. Age-related changes in the early evoked auditory potentials may also be mediated by age differences in the involvement of the PFC in attentional control [89]. Obleser and Kotz [90] suggested that age-effects on neural processing in the N1 time-window, as observed in the OAs in the current study for clear vs. degraded speech, may be reflective of increased ‘neural effort’ via resource-allocation required to decode degraded speech. Differences observed in the N1 time-window, can be interpreted as a change in cognitive strategy [53], as the listening demand increases (*clear*–*degraded*).

In contrast to OAs, YAs only showed topographical differences corresponding to the time-window of the P2 component between the two listening conditions. This might be linked to the role of the P2 time-window in allocation of attention to the salient stimulus during target detection [91, 92]. For instance, Getzmann et al. [92] showed P2-peak changes when participants focused their attention and when the target speaker was salient during a spatial listening task. The topographical differences in P2 time window observed here between clear and degraded speech for YAs but not OAs might therefore reflect the ability of YAs to selectively attend to task-relevant information (digits) and ignore the distractors (babble).

The time-wise MGFP analyses did not show any significant differences between the two conditions, differences were only evident between groups. This indicates that task difficulty and stimulus clarity did not modulate overall neural response strength, as indicated by MGFP. This finding was somewhat unexpected considering the perceptual difference between the clear and degraded conditions. Although visual inspection of the grand mean waveforms indicated larger differences at the fronto-central vertex electrodes (see also MGFP waveforms in Fig 5), these amplitude differences were not statistically significant at the MGFP level. It may also be that ERP amplitudes are not as sensitive to manipulations of the acoustic TFS in the stimuli as they are with envelope manipulations. The MGFP results were however consistent with the results of the topographical analyses which showed differences between OA and YA in the early time window. OAs had smaller overall neural response strength during the N1 time-window (with similar mean P2 amplitudes) compared to the younger participants (averaged between the two [*clear* and *degraded*] conditions, see Fig 4). This suggests that, as signal resolution (digits in noise) was reduced, at early stages of sensory processing (N1 time window) OAs allocated more of their cognitive resources (P2 time window) to process the unresolved input signal and to facilitate post-perceptual processing (P3 time window). YAs however, had a more accurate representation of the incoming bottom-up signal for both *clear* and *degraded* conditions, which enabled (early) top-down selection of salient features in the resolved signal before (later) post-perceptual processing had occurred [32]. This may be why the YAs outperformed the OAs in the number comparison task (SNR_71%_), and why YA vs. OA differences were evident across a range of latency windows. These results highlight the possibility of earlier processing deficits in OAs affecting subsequent higher-level functions of stimulus selection and response execution. Previous studies using a-priori selected electrode/s have shown increased N1 amplitudes with increasing task difficulty/speech degradation (e.g. [90]), and increased (e.g. [93]) or no changes (e.g. [94]) in N1 amplitude with aging. Aside from reporting global neural activity (MGFP & TANOVA analyses) in the current study, differences across studies can arise from the choice of reference electrode [52] as well as the stimuli and task.

Previous studies have demonstrated compensatory allocation of resources at early stages of neural processing (as indexed by increased P2 amplitude) in adults with hearing loss (mild–moderate), compared to age-matched normal-hearing controls during passive listening [95, 96]. Roque et al. [97] showed aging effects only in the P1 time window (earlier latency values), using multichannel analysis techniques and after controlling for stimulus audibility effects. The authors attributed the earlier time-window effects to the nature of the task used in the study. Our results extend these findings to active listening, controlling for confounding effects of stimulus-spectrum audibility and performance levels when assessing changes associated with neuroplasticity in OAs with a range of supra-threshold auditory processing and selective attentional abilities [51]. Unlike the Roque et al. study, where ERPs were recorded during passive listening (words in quiet), our participants performed a number comparison task in the presence of a two-talker babble. Such active listening tasks, requiring focus and response, may highlight auditory processing effects on later ERP components/time-windows (>90 msec) [98–100]. Billings et al. [99] examined P1, N1, and P2 for passive vs. active paradigms and showed attention-related effects on N1-amplitude only when listening to tones presented in noise, not in quiet. Recent fMRI results show similar brain activation patterns in younger and older individuals during a word recognition in quiet task [101]. Tasks such as that used in the current study which used competing speech babble, degraded speech acoustics and, an active listening task are more likely to elicit ERP differences that reflect aging effects on both earlier and later auditory neural processing.

### Electrophysiological differences at later latencies

Differences in neural processing in later time windows between OAs and YAs in the current study suggest that the effects of aging on early cognitive resource allocation subsequently had impacts on later time-windows of post-perceptual stimulus processing. Later time-windows beyond 400 ms include the P3b component that is often linked to processes involving cognitive processing and decision-making [67, 102]. Only the OAs showed topographical differences, with increased frontal distribution, as the listening demand increased. This is consistent with the suggestion that OAs tend to recruit more cognitive resources to address processing difficulties involving degraded bottom-up input signal [11, 103]. For instance, Wong et al. [103] showed that, when OAs listened to speech in the presence of noise, they exhibited reduced fMRI activation in the auditory cortex but increased activation in the cognitive regions of the prefrontal cortex and precuneus. However, despite this increased activation, OAs are still at a disadvantage relative to YAs as behavioral performance may be affected by reduced availability of cognitive resources [104]. This is consistent with the poorer scores obtained by the OAs for the behavioral tasks (see Fig 1) and the generally reduced ERP activity (MGFP, see Fig 4) compared to the YAs in the current study. Pergher et al. [68] similarly showed a decrease in P3 amplitude for OAs compared to YAs during a visual N-back task. They also found that P3 amplitude and alpha band activity reduced with increasing task difficulty in younger participants and alpha band activity increased in older participants due to mental fatigue. Whether the topographical differences observed in the OAs in the current study are related to fatigue, will need further investigation.

### Prefrontal source activation during degraded listening

The increased frontal distribution and enhanced frontal P3 amplitudes observed for the *degraded* condition might be a strategy adopted by the OAs to match the level of cognitive control demanded by the task [105]. The increased frontal distribution is also supported by the results of the source estimation analyses that showed activation of the left MFG for the *degraded* condition. There is complementary evidence for this from an fMRI study which also showed activation in this region when participants listened to sentences with increasing levels of acoustic degradation [106]. Recent met-analyses of fMRI studies have also suggested the involvement of areas (fronto-parietal and insula regions) beyond the auditory cortex during effortful speech perception [107]. The meta-analyses also revealed that patterns of activation differed depending on the effortful listening condition (spectral degradation vs speech in noise). A combined ERP/fMRI study showed activation in the right MFG during visual-target-processing (P3b) for older compared to younger participants [67]; the difference in MFG laterality compared to the current study may be attributable to task differences such as the auditory and linguistic demands of the task used in the current study. Although literature (see meta-analyses [107] shows some variability in the exact location of activation, activity in the left prefrontal regions has been associated with resolving degraded speech input (e.g. [108]).

The current study demonstrated an association between left prefrontal cortex activation and poorer speech recognition abilities in OAs. This pattern is consistent with previous research that has shown age-related enhancement of frontal P3 ERPs associated with poorer performance on neuropsychological tests of frontal lobe function [109]. Similarly, Reuter et al. [49] showed that a subset of OAs with increased frontal P3 distribution exhibited poorer performance on a visual selective attention task. The authors suggested that such parietal vs. frontal ERP distribution helped differentiate subgroups of OAs with less efficient processing. Our study extends these findings using source analyses and shows exploratory evidence (moderate correlation) for inefficient processing with increasing listening demands through the recruitment of the left MFG in OAs with poorer scores on the listening task. It is important to note, however, that individual differences in performance and recruitment of the prefrontal cortex have been linked to brain maintenance (or inefficient processing) rather than compensatory processes associated with aging [110, 111]. In particular, Morcom and Henson [111] showed that increased frontal recruitment (via fMRI) was task dependent and that it did not necessarily facilitate (or improve) performance with increasing age. This finding challenges existing compensatory theories (e.g. PASA) that suggest a generalized recruitment of prefrontal cortex to maintain optimal performance in all older individuals.

The results observed here are also consistent with recent longitudinal studies where only participants with declining cognitive functions showed *increasing* prefrontal (including left MFG) activation (baseline < follow-up) compared to those with stable performance on behavioral tasks [112, 113]. The observed prefrontal activation in the current study, hence, cannot be generalized as a typical finding in the aging population. Rather, this occurred only during the most effortful listening condition (*degraded*) and in poorer performers who required higher SNRs to process degraded speech. In addition, we also showed supporting evidence for increasing posterior to anterior shift in poorer-performing OAs compared to high-performing OAs and YAs (see Fig 7(d)), thus, it is highly likely that our sample included a mixture of high-functioning older individuals as well as those with poorer cognitive function. Also, OAs in the poorer-performing group had poorer TFS1 and ANT-composite scores (*M*_*TFS1*_ 30.8, *M*_*ANT*_ 39.7) compared to the good-performing OAs (*M*_*TFS1*_ 18.4, *M*_*ANT*_ 42.2). Two previous studies that subdivided older participants based on their memory performance relative to IQ scores, showed similar evidence for more frontal activity (P3 time window) in poor performing OAs compared to high performers and younger adults [114, 115]. Hence, the frontal distribution in the P3 time-window, especially for the poor performing OAs in this study might be associated with less efficient recruitment of neural resources during task performance [109, 111]. Differences in network efficiency could be investigated using graph theoretical methods in future research (e.g. [48]), comparing OAs with good and poor performance to determine whether increased network efficiency in OAs results in task performance that is comparable to younger adults.

## Conclusions and future directions

Overall, this study provides important insight into neural processing associated with age-related speech-in-noise difficulties. Our findings support evidence that age-related speech processing deficits are a consequence of early auditory processing deficits [116]. Specifically, older individuals with deficits in early stages of auditory neural processing display activation of brain regions outside the core speech areas (see Fig 4 in [17]) during later stages of *degraded* speech processing. The regression analyses suggested that task performance depended on supra-threshold auditory processing abilities (TFS1). Thus, hearing loss/audibility compensation via hearing aid amplification might not be the only determinant factor for successful speech recognition in noise by OAs with/without hearing loss [117]. Also, OAs with poor supra-threshold temporal processing abilities have deficits at the subcortical level (e.g. [29]). Hence a future study examining subcortical and cortical activation could demonstrate whether OA with subcortical processing deficits show prefrontal source activation, as observed in the current study.

Despite the findings and methodological features of this study, the main limitation was the inclusion of participants with hearing loss in the OA group. The stimulus spectrum-audibility was adjusted across the OAs and stimuli were presented at similar intensity levels between groups. Although this minimized audibility effects, the contribution of peripheral hearing loss cannot be completely ruled out as OAs typically have some form of hearing loss or poorer hearing thresholds at frequencies beyond 8 kHz [26, 118]. We note that audibility adjustments made according to individual audiograms may not be sufficient to compensate for the hearing loss observed here and can also have potentials effects on cortical responses that is yet to be completely understood [31, 51]. We however did not observe a correlation between task performance and high frequency hearing loss in the participants included in this study and hence believe findings to be relevant. Also, while it is desirable to compare OAs with and without hearing loss, it is quite challenging to recruit participants matched in age, hearing thresholds, etiology, and duration of hearing loss.

Although we used a within-subject design to control for individual differences, variables such as handedness, visual acuity, linguistic background, and neuropsychological profile are potential confounds (e.g [119]) that could affect YA vs. OA and other group comparisons. And hence, these variables should be examined or controlled for in a larger study with a more representative sample.

While the number of electrodes used here gave us valuable insights about the differences in the underlying source activation, a more localized analysis of the brain activation would need a larger number of electrodes to avoid mislocalizations and blurring effects. A longitudinal study design with the addition of high-density EEG recordings to reduce source localization errors [63], may allow a future study to better understand neural mechanisms underlying age-related listening difficulties, particularly those associated with cognitive (e.g. mild cognitive impairment) as well as sensory deficits.

## Supporting information

S1 File Regression analyses for behavioural data across older and younger adults. (DOCX)
