# Supplementary material for: Effects of aging on neural processing during an active listening task
Source: PLoS One. 2022 Sep 7;17(9):e0273304. doi: 10.1371/journal.pone.0273304 (PMC9451064; doi:10.1371/journal.pone.0273304)
Supplement: S1 File — (DOCX) [file pone.0273304.s001.docx]

**Supporting Information**

**Regression analyses for behavioural data across older and younger adults**

Table 1. Stepwise regression of SNR_71%_ ~ ANT + TFS1 for the older adults

| **Model Summary** | | | | | | | | | |
| --- | --- | --- | --- | --- | --- | --- | --- | --- | --- |
| Model | R | R Square | Adjusted R Square | Std. Error of the Estimate | Change Statistics | | | | |
|  |  |  |  |  | R Square Change | F Change | df1 | df2 | Sig. F Change |
| 1 | .421^a^ | .177 | .148 | 1.67859 | .177 | 6.023 | 1 | 28 | .021 |
| a. Predictors: (Constant), TFS1 | | | | | | | | | |

| **ANOVA^a^** | | | | | | |
| --- | --- | --- | --- | --- | --- | --- |
| Model | | Sum of Squares | df | Mean Square | F | Sig. |
| 1 | Regression | 16.972 | 1 | 16.972 | 6.023 | .021^b^ |
|  | Residual | 78.895 | 28 | 2.818 |  |  |
|  | Total | 95.867 | 29 |  |  |  |
| a. Dependent Variable: SNR_71%_ | | | | | | |
| b. Predictors: (Constant), TFS1 | | | | | | |

| **Coefficients^a^** | | | | | | | | | | | |
| --- | --- | --- | --- | --- | --- | --- | --- | --- | --- | --- | --- |
| Model | | Unstandardized Coefficients | | Standardized Coefficients | t | Sig. | Correlations | | | Collinearity Statistics | |
|  |  | B | Std. Error | Beta |  |  | Zero-order | Partial | Part | Tolerance | VIF |
| 1 | (Constant) | .853 | .653 |  | 1.307 | .202 |  |  |  |  |  |
|  | O_TFS1 | .057 | .023 | .421 | 2.454 | .021 | .421 | .421 | .421 | 1.000 | 1.000 |
| a. Dependent Variable: SNR_71%_ | | | | | | | | | | | |

**Excluded variables**

ANT

Significance = p > 0.05

Table 2. Stepwise regression of SNR_71%_ ~ ANT + TFS1 for the younger adults

| **Model Summary** | | | | | | | | | |
| --- | --- | --- | --- | --- | --- | --- | --- | --- | --- |
| Model | R | R Square | Adjusted R Square | Std. Error of the Estimate | Change Statistics | | | | |
|  |  |  |  |  | R Square Change | F Change | df1 | df2 | Sig. F Change |
| 1 | .446^a^ | .199 | .155 | 1.47334 | .199 | 4.481 | 1 | 18 | .048 |
| a. Predictors: (Constant), ANT | | | | | | | | | |

| **ANOVA^a^** | | | | | | |
| --- | --- | --- | --- | --- | --- | --- |
| Model | | Sum of Squares | df | Mean Square | F | Sig. |
| 1 | Regression | 9.727 | 1 | 9.727 | 4.481 | .048^b^ |
|  | Residual | 39.073 | 18 | 2.171 |  |  |
|  | Total | 48.800 | 19 |  |  |  |
| a. Dependent Variable: SNR_71%_ | | | | | | |
| b. Predictors: (Constant), ANT | | | | | | |

| **Coefficients^a^** | | | | | | | | | | | |
| --- | --- | --- | --- | --- | --- | --- | --- | --- | --- | --- | --- |
| Model | | Unstandardized Coefficients | | Standardized Coefficients | t | Sig. | Correlations | | | Collinearity Statistics | |
|  |  | B | Std. Error | Beta |  |  | Zero-order | Partial | Part | Tolerance | VIF |
| 1 | (Constant) | 1.161 | 1.254 |  | .926 | .367 |  |  |  |  |  |
|  | Y_ANT | -.066 | .031 | -.446 | -2.117 | .048 | -.446 | -.446 | -.446 | 1.000 | 1.000 |
| a. Dependent Variable: SNR_71%_ | | | | | | | | | | | |

**Excluded variables**

TFS1

Significance = p > 0.05

Table 3. Hierarchical regression of SNR_71%_ ~ ANT + TFS1 + thresholds for the older adults

| **Variables Entered/Removed^a^** | | | |
| --- | --- | --- | --- |
| Model | Variables Entered | Variables Removed | Method |
| 1 | Hearing thresholds, TFS1, ANT^b^ | . | Enter |
| 2 | .^b^ | Hearing Thresholds^c^ | Remove |
| 3 | .^b^ | TFS1^c^ | Remove |
| a. Dependent Variable: SNR_71%_ | | | |
| b. All requested variables entered. | | | |
| c. All requested variables removed. | | | |

| **Model Summary** | | | | | | | | | |
| --- | --- | --- | --- | --- | --- | --- | --- | --- | --- |
| Model | R | R Square | Adjusted R Square | Std. Error of the Estimate | Change Statistics | | | | |
|  |  |  |  |  | R Square Change | F Change | df1 | df2 | Sig. F Change |
| 1 | .453^a^ | .205 | .113 | 1.71202 | .205 | 2.236 | 3 | 26 | .108 |
| 2 | .446^b^ | .199 | .140 | 1.68623 | -.006 | .192 | 1 | 26 | .665 |
| 3 | .135^c^ | .018 | -.017 | 1.83354 | -.181 | 6.106 | 1 | 27 | .020 |
| a. Predictors: (Constant), Hearing thresholds, TFS1, ANT | | | | | | | | | |
| b. Predictors: (Constant), TFS1, ANT | | | | | | | | | |
| c. Predictors: (Constant), ANT | | | | | | | | | |

| **ANOVA^a^** | | | | | | |
| --- | --- | --- | --- | --- | --- | --- |
| Model | | Sum of Squares | df | Mean Square | F | Sig. |
| 1 | Regression | 19.660 | 3 | 6.553 | 2.236 | .108^b^ |
|  | Residual | 76.207 | 26 | 2.931 |  |  |
|  | Total | 95.867 | 29 |  |  |  |
| 2 | Regression | 19.096 | 2 | 9.548 | 3.358 | .050^c^ |
|  | Residual | 76.771 | 27 | 2.843 |  |  |
|  | Total | 95.867 | 29 |  |  |  |
| 3 | Regression | 1.734 | 1 | 1.734 | .516 | .479^d^ |
|  | Residual | 94.132 | 28 | 3.362 |  |  |
|  | Total | 95.867 | 29 |  |  |  |
| a. Dependent Variable: SNR_71%_ | | | | | | |
| b. Predictors: (Constant), Hearing thresholds, TFS1, ANT | | | | | | |
| c. Predictors: (Constant), TFS1, ANT | | | | | | |
| d. Predictors: (Constant), ANT | | | | | | |

| **Coefficients^a^** | | | | | | |
| --- | --- | --- | --- | --- | --- | --- |
| Model | | Unstandardized Coefficients | | Standardized Coefficients | t | Sig. |
|  |  | B | Std. Error | Beta |  |  |
| 1 | (Constant) | 1.428 | .937 |  | 1.523 | .140 |
|  | TFS1 | .058 | .024 | .427 | 2.441 | .022 |
|  | ANT | -.017 | .018 | -.181 | -.954 | .349 |
|  | Hearing thresholds | .315 | .717 | .083 | .439 | .665 |
| 2 | (Constant) | 1.414 | .923 |  | 1.532 | .137 |
|  | TFS1 | .058 | .023 | .426 | 2.471 | .020 |
|  | ANT | -.014 | .016 | -.149 | -.864 | .395 |
| 3 | (Constant) | 2.789 | .801 |  | 3.484 | .002 |
|  | ANT | -.013 | .018 | -.135 | -.718 | .479 |
| a. Dependent Variable: SNR_71%_ | | | | | | |
